# Role of Immunoglobulin A in COVID-19 and Influenza Infections

**DOI:** 10.3390/vaccines11111647

**Published:** 2023-10-27

**Authors:** Rohit Tyagi, Srijani Basu, Atika Dhar, Suman Gupta, Sneh Lata Gupta, Rishi K. Jaiswal

**Affiliations:** 1 College of Veterinary Medicine, Huazhong Agricultural University, Wuhan 430070, China; rhtyagi@webmail.hzau.edu.cn; 2 Department of Medicine, Weill Cornell Medicine, New York, NY 10065, USA; srijanibasu@gmail.com; 3 National Institutes of Health, Bethesda, MD 20892, USA; atika.dhar@nih.gov; 4 Department of Medicine, Cedars-Sinai Medical Center, Los Angeles, CA 90048, USA; 5 National Institute of Immunology, New Delhi 110067, India; 6 Department of Cancer Biology, Cardinal Bernardin Cancer Center, Loyola University Chicago, Stritch School of Medicine, Maywood, IL 60153, USA

## 1. Introduction

Immunoglobulin A (IgA) is critical in the immune response against respiratory infections like COVID-19 and influenza. IgA is one of the five main classes of immunoglobulins or antibodies, that is primarily found in mucosal tissues, including the respiratory and gastrointestinal tracts [1]. Here, we will explore the role of IgA in COVID-19 and influenza infections. Both COVID-19 and flu are respiratory viral diseases, and mucosal immunity is important in generating a protective response against them. An important question, therefore, is whether antibody cross-reactivity between diverse viral strains is a cause of pre-existing immunity to emergent viral variants and when individuals are vaccinated against a new viral strain of SARS-CoV-2, does it have the potential to “back-boost” their existing immunity against previously circulating strains?

Here, we shed light on some of these interesting propositions.

In COVID-19 infections, IgA is a key component of the adaptive immune response to SARS-CoV-2, the virus responsible for COVID-19. IgA antibodies specific to the virus are produced by plasma cells in the mucosal-associated lymphoid tissues (MALTs), such as the tonsils, and are secreted into the respiratory tract, where they play several important roles such as in mucosal defense, preventing entry of the virus, and reducing transmission. One report tested influenza- and COVID-19-specific antibodies post vaccination and found antigen-specific IgA antibodies in the saliva [2]. In the early response to acute COVID-19 infection, the dominant neutralizing antibodies formed were of the IgA isotype and were detected in saliva, nasopharyngeal swabs, and serum for at least one month [3]. In fact, the absence of SARS-CoV-2-specific IgA antibody could be a cause of vaccine failure, exacerbated COVID-19, and possible prolonged virus shedding in patients with primary antibody deficiency and selective IgA deficiency [4].

**Figure 1:**
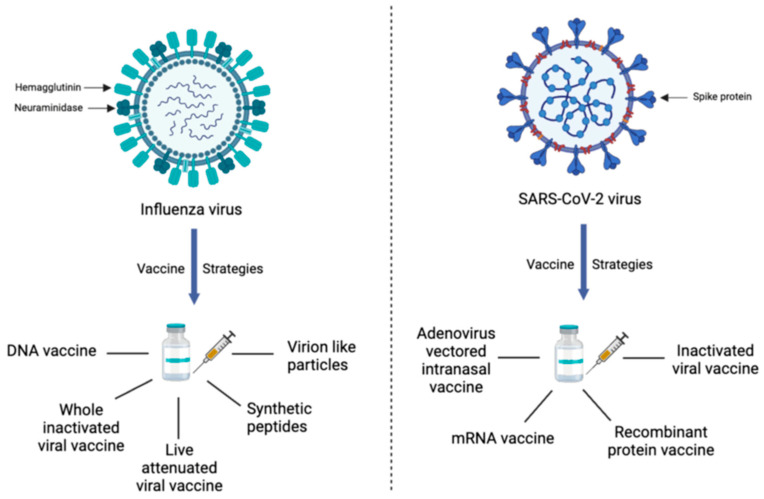
Various vaccine strategies being developed against Influenza and SARS-CoV-2. Both virues have distinct surface protein structure which have been used to detect and classify various viral strains. The current vaccinestrategies include utilizing different approaches to elicit prolonged neutralizing antibodytiters.

IgA also plays an important role in the immune response to influenza viruses. Like COVID-19, IgA in the respiratory tract contributes to defenses against influenza infection. Repeated influenza vaccines enhance IgG and IgA levels and boost their previous immunity. In the case of COVID-19 vaccine recipients, both IgG and IgA antibodies increased in serum after infection, but the IgA level was similar, irrespective of previous infection exposures [5,6,7]. COVID-19 vaccine-induced IgG and IgA antibody generation in serum also depends on the vaccine type since mRNA vaccines (Moderna > Pfizer) generate a higher immune response than DNA plasmid vector-based (AstraZeneca > Sputnik-V > Johnson and Johnson) and attenuated virus (Sinopharm) vaccines [8,9]. The recent seasonal influenza vaccine being administered is based on an inactivated virus. All COVID-19 vaccinations so far are based on the spike components of the virus, while influenza vaccines utilize two surface glycoproteins, namely hemagglutinin (HA) and neuraminidase (NA) [10]. The various strategies for vaccine development against influenza and COVID-19 have been summarized in Figure 1.

Reports on the simultaneous administration of the COVID-19 mRNA booster and the flu vaccine showed that systemic reaction increased by 8–11% compared to only the COVID-19 mRNA booster alone. Thus, overall reactogenicity and SARS-CoV-2-binding titers either remained similar or enhanced, with no adverse side effects [11,12]. There is another report showing that the administration of a flu vaccine decreases the COVID-19 disease severity [13]. However, a study on transgenic mice revealed that prior influenza infection increases the vulnerability to COVID-19 disease severity, morbidity, and mortality. These data also reflect the necessity of influenza vaccination to prevent severe disease outcomes for COVID-19 and/or influenza [14]. Nasopharyngeal-associated lymphoid tissue (NALT) is a major location of immune modulation by pathogens that enter the host via the nasal mucosa and for nasal vaccines [15]. Nasally administered vaccines show improved mucosal as well as systemic immunity that makes them a promising bet for the design of novel vaccines and immunotherapeutic interventions against pulmonary pathogens. In addition to the generation of a systemic neutralizing antibody response and T cell-based immunity against a majority of Omicron subvariants, one nasal vaccine generated spike-specific IgA in the respiratory mucosa and T cell immune responses focused in the NALT [16]. When the COVID-19 pandemic began, the vaccine’s notion had been extensively investigated with regard to the Ebola and H5N1 influenza viruses [17]. Utilizing the design formulated in vaccination studies against Ebola and H5N1 influenza viruses, a nasal vaccine study utilized live vaccination based on an attenuated avian paramyxovirus type 3 (APMV3). In another nasal vaccine-based study, the SARS-CoV-2 spike protein was introduced into the lungs through a weaker variant of a bovine/human parainfluenza virus (B/HPIV3) [18].

For the flu virus, FluMist Quadrivalent is the only nasal spray vaccine available in the U.S. [19]. In an important study, an intranasal NS1-deleted influenza virus vectored COVID-19 vaccine candidate called dNS1-RBD was administered to a hamster model and successfully activated protective immunity [20]. Compared to the wild-type H1N1 influenza strain (A/PR8/NSfull), the NS1-truncated virus (A/PR8/NS124) induced a stronger effector T cell response through intranasal vaccination. The resulting response provided protection against the lethal heterologous A/Aichi/2/68 (H3N2) influenza virus through substantially lowering inflammation and pathology without reducing viral load [21].

Investigators in Beijing used a weakened influenza virus as a vector for the SARS-CoV-2 RBD (receptor-binding domain) and labeled it as CA4-dNS-nCoV-RBD or dNS1-RBD. In studies using BALB/c mice, a vaccine regimen involving both a prime and a boost demonstrated the ability to stimulate RBD-specific T cell responses localized within alveolar tissues. Additionally, it induces ample amounts of RBD-specific IgA and IgG antibodies in the lung bronchoalveolar lavage (BAL) fluid. Furthermore, when these mice were exposed to SARS-CoV-2 Omicron in a controlled experiment, the dNS1-RBD vaccine provided protection by avoiding chronic disease and lowering the lung’s pathogen load [22].

In a phase 2 clinical trial that followed a randomized, double-blind, placebo-controlled design, approximately 40% of dNS1-RBD vaccine recipients developed systemic T cell responses, while fewer than 22% developed RBD-specific IgG responses. However, the vaccine’s localized MALT responses were generated in only 13% of individuals, which was a weak sIgA response, even though the response was well-tolerated [23].

A nasal vaccine for COVID-19 was approved in 2021 by Iran (Razi Vaccine and Serum Research Institute in Karaj). In 2022, CanSino Biologics in Tianjin, China, and Bharat Biotech in Hyderabad, India, launched their respective nasal vaccines administered as mist and nasal drops, respectively. Russia also approved the use of an intranasal form of Sputnik V in 2022 [24]. The MV-014-212 vaccine is a weakened human respiratory syncytial virus intranasal vaccine designed by Meissa Vaccines to express SARS-CoV-2 spike protein. It generated a boost in the immune response during the non-human primate trials. Specifically, it led to approximately 8 times higher levels of nasal IgA and 2 times higher levels of serum IgG antibodies targeting the spike protein [25]. The viral titers in nasal swabs and BAL (bronchoalveolar lavage) samples from the vaccinated animals were significantly decreased (1000 fold) after the virus challenge, even though a direct analysis of the relationship between mucosal IgA and protection in vaccinated animals was not observed. Then, in the subsequent phase I clinical trials, even a single dose of the vaccine triggered a significant amount of nasal IgA response in comparison to individuals who encounter actual SARS-CoV-2 infections.

## 2. Challenges to the Development of Mucosal Vaccines

The effectiveness of mucosal vaccines for COVID-19 faces several challenges. Firstly, a significant portion of the global population has already been exposed to the virus through natural infection, potentially possessing some degree of mucosal immunity. This raises questions about the need for mucosal vaccines when much of the population may already have some level of protection. Additionally, the durability of mucosal immune responses is a concern, as studies have shown that airway IgA, a key component of mucosal immunity, may decrease rapidly between 3 and 9 months after recovering from COVID-19, especially in mild to moderate cases [26].

However, there is some optimism based on pre-clinical models and the concept of “hybrid immunity”, where mucosal vaccination in individuals with existing immunity from other forms of immunization can enhance mucosal protection. This approach may extend systemic immunity to the mucosa without the risk of severe clinical outcomes, even in cases of breakthrough infections like those seen with the Omicron variant [27]. Another consideration is the experience with live-attenuated influenza vaccines (LAIVs), which can induce both systemic and mucosal immunity but have not consistently outperformed traditional injectable vaccines in terms of effectiveness. Challenges with LAIVs include issues related to viral production and host immunity against the viral vector [28,29,30].

To address these challenges, alternative mucosal vaccine platforms are being explored, such as synthetic mRNA, recombinant proteins, and various delivery systems [31]. These approaches aim to improve the immunogenicity of mucosal vaccines while maintaining safety. Additionally, multi-dose regimens are being considered to enhance the immune response, particularly through intranasal delivery, which is a practical route for mucosal vaccination. However, these strategies are still in the experimental stage and have mostly been tested through traditional injection methods. While there are challenges associated with mucosal vaccines for COVID-19, there is ongoing research and development aimed at optimizing their effectiveness and safety.

## 3. Conclusions

In conclusion, by taking into consideration the above studies, we can infer that intranasal vaccines have a good potential to be used as booster doses to counter seasonal reoccurrence of both SARS-CoV-2 and influenza viruses. Mucosal IgA provides a more localized intervention to retard virus attachment and transcription in the host cells, making them an appropriate candidate for localized restriction of pathogens. Also, intranasal vaccines are user-friendly and can be safely applied which can lower the high cost of vaccination programs globally. IgA plays a crucial role in the defense against both COVID-19 and influenza infections by providing mucosal immunity, neutralizing the viruses, and reducing their transmission. Understanding the role of IgA in these infections is vital for developing effective vaccines and therapeutic strategies.
